# Crystal structure of 2,4-di­amino-7-(hydroxy­meth­yl)pteridin-1-ium nitrate

**DOI:** 10.1107/S2056989015008397

**Published:** 2015-05-07

**Authors:** Palaniyappan Sivajeyanthi, Kasthuri Balasubramani, Muthaiah Jeevaraj, Kaliyaperumal Thanigaimani, Nuridayanti Che Khalib, Ibrahim Abdul Razak

**Affiliations:** a Department of Chemistry, Government Arts College (Autonomous), Thanthonimalai, Karur 639 005, Tamil Nadu, India; b School of Physics, Universiti Sains Malaysia, 11800 USM, Penang, Malaysia

**Keywords:** crystal structure, pteridine, 2,4-di­amino­pteridinium, pteridin-1-ium nitrate, hydrogen bonding, ring motifs

## Abstract

In the crystal of the title mol­ecular salt, C_7_H_9_N_6_O^+^·NO_3_^−^, the cations and anions are linked *via* N—H⋯O and O—H⋯O hydrogen bonds, forming sheets parallel to (100). Within the sheets there are numerous hydrogen-bonding ring motifs.

## Related literature   

For background to and the biological activity of pteridine derivatives, see: Benkovic Annu (1980 ▸); Blakeley (1969 ▸); Van Beelen *et al.* (1984 ▸); Dolphin (1980 ▸); Pfleiderer (1982 ▸); Blakely & Cocco (1985 ▸); Pfleiderer & Taylor (1964 ▸); Müller *et al.* (1991 ▸); Weinstock *et al.* (1968 ▸). For related structures, see: Kuyper (1990 ▸); Schwalbe & Williams (1986 ▸); Robertson *et al.* (1998 ▸). For hydrogen-bond motifs, see: Etter (1990 ▸); Bernstein *et al.* (1995 ▸); Allen *et al.* (1998 ▸).
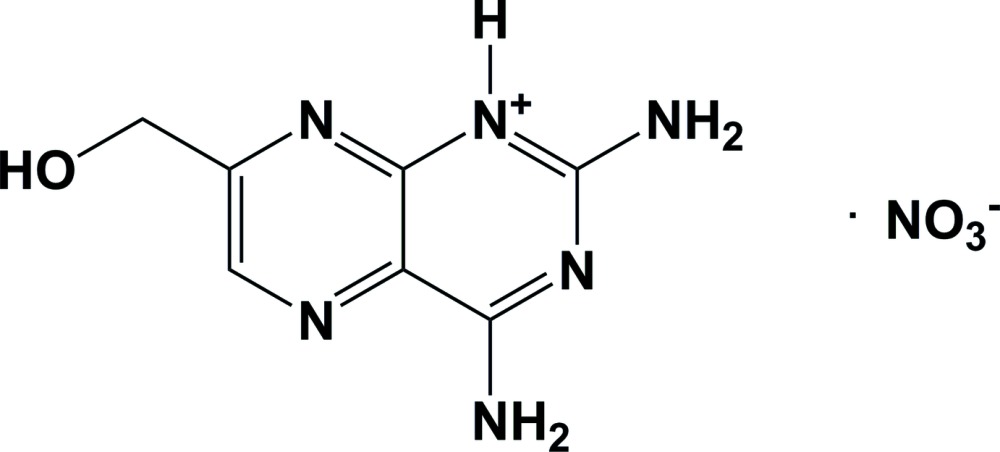


## Experimental   

### Crystal data  

C_7_H_9_N_6_O^+^·NO_3_^−^
*M* *_r_* = 255.21
Orthorhombic, 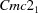

*a* = 6.4060 (17) Å
*b* = 14.960 (6) Å
*c* = 10.867 (3) Å
*V* = 1041.4 (6) Å^3^
*Z* = 4
Mo *K*α radiation
μ = 0.14 mm^−1^
*T* = 294 K
0.27 × 0.10 × 0.07 mm

### Data collection  

Bruker SMART APEXII Duo CCD area-detector diffractometer
Absorption correction: multi-scan (*SADABS*; Bruker, 2009 ▸) *T*_min_ = 0.964, *T*_max_ = 0.991
2866 measured reflections
862 independent reflections
720 reflections with *I* > 2σ(*I*)
*R*_int_ = 0.028

### Refinement  

*R*[*F* ^2^ > 2σ(*F* ^2^)] = 0.030
*wR*(*F* ^2^) = 0.074
*S* = 1.03
862 reflections
127 parameters
1 restraint
H atoms treated by a mixture of independent and constrained refinement
Δρ_max_ = 0.09 e Å^−3^
Δρ_min_ = −0.16 e Å^−3^

### 

Data collection: *APEX2* (Bruker, 2009 ▸); cell refinement: *SAINT* (Bruker, 2009 ▸); data reduction: *SAINT*; program(s) used to solve structure: *SHELXS97* (Sheldrick, 2008 ▸); program(s) used to refine structure: *SHELXL97* (Sheldrick, 2008 ▸); molecular graphics: *SHELXTL* (Sheldrick, 2008 ▸); software used to prepare material for publication: *SHELXTL* and *PLATON* (Spek, 2009 ▸).

## Supplementary Material

Crystal structure: contains datablock(s) global, I. DOI: 10.1107/S2056989015008397/su5127sup1.cif

Structure factors: contains datablock(s) I. DOI: 10.1107/S2056989015008397/su5127Isup2.hkl

Supporting information file. DOI: 10.1107/S2056989015008397/su5127Isup3.cml

**Fig. 1.**
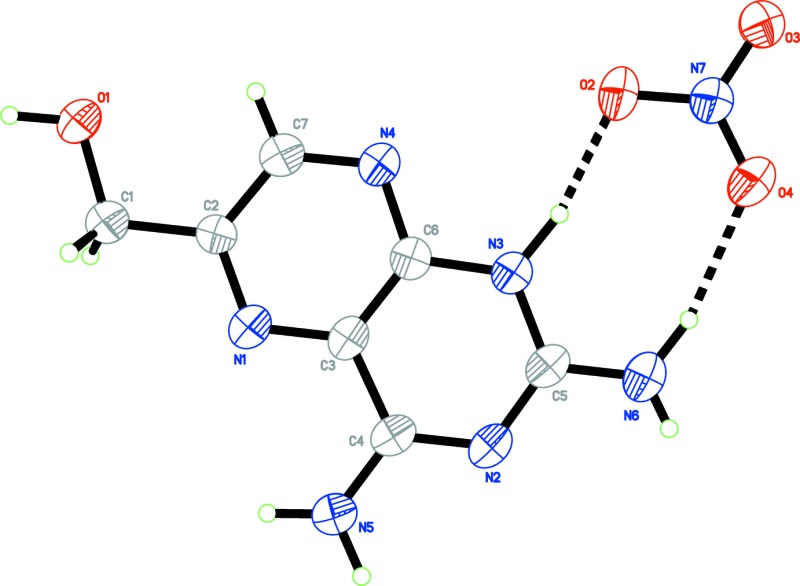
The molecular structure of the title salt, with atom labelling. Displacement ellipsoids are drawn at the 50% probability level. The N-H···O hydrogen bonds are shown as dashed lines (see Table 1 for details).

**Fig. 2.**
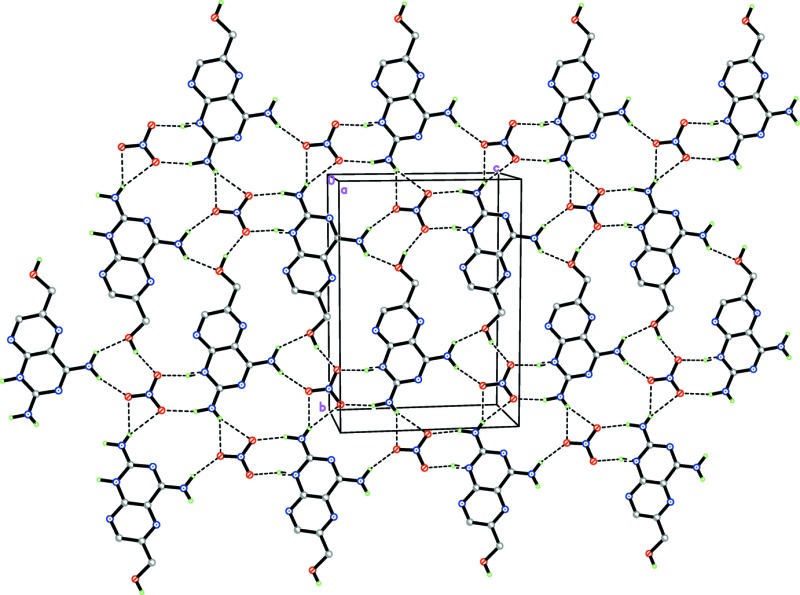
A view along the a axis of the crystal packing of the title molecular salt. The N-H···O hydrogen bonds are shown as dashed lines (see Table 1 for details).

. DOI: 10.1107/S2056989015008397/su5127fig1.tif
The mol­ecular structure of the title salt, with atom labelling. Displacement ellipsoids are drawn at the 50% probability level. The N-H⋯O hydrogen bonds are shown as dashed lines (see Table 1 for details).

a . DOI: 10.1107/S2056989015008397/su5127fig2.tif
A view along the *a* axis of the crystal packing of the title mol­ecular salt. The N-H⋯O hydrogen bonds are shown as dashed lines (see Table 1 for details).

CCDC reference: 1062258

Additional supporting information:  crystallographic information; 3D view; checkCIF report

## Figures and Tables

**Table 1:** Hydrogen-bond geometry (, )

*D*H*A*	*D*H	H*A*	*D* *A*	*D*H*A*
N3H1*N*3O2	1.03(5)	1.82(5)	2.831(5)	167(4)
N6H2*N*6O4	0.98(3)	2.06(3)	3.019(6)	167(3)
O1H1*O*1O2^i^	0.95(6)	1.93(6)	2.846(4)	163(6)
N5H1*N*5O3^ii^	0.92(4)	2.09(4)	2.983(6)	164(4)
N5H2*N*5O1^i^	0.91(5)	2.15(5)	2.913(5)	141(4)
N6H1*N*6O3^iii^	0.78(5)	2.35(4)	3.015(5)	145(5)
N6H1*N*6O4^iii^	0.78(5)	2.44(5)	3.190(6)	162(5)

Symmetry codes: (i) 
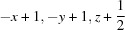
; (ii) 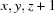; (iii) 
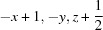
.

## supplementary crystallographic information

### S1. Structural commentary

Pteridine derivatives are found as the core structure of folic acid flavin adenine dinucleotide (FAD) and function as cofactors for enzymes involved in hy­droxy­lation (Benkovic & Annu,1980) and methyl transfer (Blakeley, 1969; Van Beelen *et al.*, 1984), as redox mediators (Dolphin, 1980) and as pigments for eyes and wings in certain insects (Pfleiderer, 1982). Variation of the substituents on the pteridine core of folates has provided synthetic anti­cancer drugs (Blakely & Cocco, 1985; Pfleiderer & Taylor, 1964). Pterdines are metabolites formed by a bicyclic pyrimidine-pyrazine moiety that occurs in a wide range of living systems and contributes in relevant biological functions (Muller *et al.*, 1991). Pteridine derivatives have good diuretic activity and are also used as simple models for therapeutically valuable anti­folate drugs (Weinstock *et al.*, 1968). In order to study potential hydrogen bonding inter­actions, the title compound was synthesized and we report herein on its crystal structure.

The title molecular salt, Fig. 1, consists of a 2,4-di­amino-7-(hy­droxy­methyl)­pteridinum cation and a nitrate anion. During the synthesis of the molecular salt a proton was transferred from the hydroxyl group of nitric acid to atom N3 of the pteridine ring. The pteridine ring system (C1—C7/N1—N6/O1) is planar with a maximum deviation of 0.001 (1) Å for all the non H atoms. The bond lengths and angles are close to those found for similar compounds, *viz.* 2,4-di­amino-6,7-di­methyl­pteridine hydro­chloride monohydrate (Schwalbe & Williams, 1986) and triamterenium tetra­phenyl­borate aceto­nitrile solvate (Robertson *et al.*, 1998).

In the crystal, Fig. 2, the protonated N3 atom and the protonated 2-amino group (N6) are hydrogen-bonded to the nitrate O atoms (O2 and O4) *via* a pair of N3—H1N3···O2 and N6—H2N6···O4 hydrogen bonds, forming an *R*_2_^2^(8) ring motif (Bernstein *et al.*, 1995). This type of inter­action is similar to the carboxyl­ate-trimethoprim inter­action observed in the trimethoprim cation-di­hydro­folate redu­ctase complex (Kuyper, 1990) and to the cyclic hydrogen bonded motif observed in many organic crystal structures (Allen *et al.*, 1998). An *R*_1_^2^(4) ring motif indicates a bifurcated hydrogen bond formed by N6–H1N6 to the two acceptors (O3 and O4). The 4-amino group and the hydroxyl group form hydrogen bonds with the O atoms of the nitrate ion leading to an *R*_3_^3^(8) ring. The three center and bifurcated hydrogen bonds and fork like inter­action form an *R*_4_^4^(12) ring. Two *R*_3_^3^(8) motifs and an *R*_2_^2^(8) ring motif generate a new *R*_3_^2^(18) ring motif. The above inter­actions lead to the formation a two dimensional network parallel to the *bc* plane (Table 1 and Fig. 2).

### S2. Synthesis and crystallization

A few drops of nitric acid were added to a hot methanol solution (20 ml) of 2,4-di­amino-6-(hy­droxy­methyl)­pteridine (43 mg, Aldrich) which had been warmed over a heating magnetic stirrer hotplate for a few minutes. The resulting solution was allowed to cool slowly at room temperature and crystals of the title molecular salt appeared after a few days.

### S3. Refinement

Crystal data, data collection and structure refinement details are summarized in Table 2. The O– and N-bound H atoms were located in a difference Fourier map and freely refined (O–H = 0.94 (6) Å and N–H = 0.78 (5)– 1.03 (5) Å). The C-bound H atoms were positioned geometrically (C–H = 0.93–0.97 Å) and refined using a riding model with *U*_iso_(H) = 1.2 *U*_eq_(C).

### Crystal data

**Table:**

C_7_H_9_N_6_O^+^·NO_3_^−^	*F*(000) = 528
*M**_r_* = 255.21	*D*_x_ = 1.628 Mg m^−^^3^
Orthorhombic, *C**m**c*2_1_	Mo *K*α radiation, λ = 0.71073 Å
Hall symbol: C 2c -2	Cell parameters from 1083 reflections
*a* = 6.4060 (17) Å	θ = 2.7–24.6°
*b* = 14.960 (6) Å	µ = 0.14 mm^−^^1^
*c* = 10.867 (3) Å	*T* = 294 K
*V* = 1041.4 (6) Å^3^	Block, bronze
*Z* = 4	0.27 × 0.10 × 0.07 mm

### Data collection

**Table:**

Bruker SMART APEXII Duo CCD area-detector diffractometer	862 independent reflections
Radiation source: fine-focus sealed tube	720 reflections with *I* > 2σ(*I*)
Graphite monochromator	*R*_int_ = 0.028
φ and ω scans	θ_max_ = 25.0°, θ_min_ = 2.7°
Absorption correction: multi-scan (*SADABS*; Bruker, 2009)	*h* = −5→7
*T*_min_ = 0.964, *T*_max_ = 0.991	*k* = −17→17
2866 measured reflections	*l* = −8→12

### Refinement

**Table:**

Refinement on *F*^2^	Primary atom site location: structure-invariant direct methods
Least-squares matrix: full	Secondary atom site location: difference Fourier map
*R*[*F*^2^ > 2σ(*F*^2^)] = 0.030	Hydrogen site location: inferred from neighbouring sites
*wR*(*F*^2^) = 0.074	H atoms treated by a mixture of independent and constrained refinement
*S* = 1.03	*w* = 1/[σ^2^(*F*_o_^2^) + (0.0382*P*)^2^ + 0.1935*P*] where *P* = (*F*_o_^2^ + 2*F*_c_^2^)/3
862 reflections	(Δ/σ)_max_ < 0.001
127 parameters	Δρ_max_ = 0.09 e Å^−^^3^
1 restraint	Δρ_min_ = −0.16 e Å^−^^3^

### Special details

**Table:**

Geometry. All e.s.d.'s (except the e.s.d. in the dihedral angle between two l.s. planes) are estimated using the full covariance matrix. The cell e.s.d.'s are taken into account individually in the estimation of e.s.d.'s in distances, angles and torsion angles; correlations between e.s.d.'s in cell parameters are only used when they are defined by crystal symmetry. An approximate (isotropic) treatment of cell e.s.d.'s is used for estimating e.s.d.'s involving l.s. planes.
Refinement. Refinement of *F*^2^ against ALL reflections. The weighted *R*-factor *wR* and goodness of fit *S* are based on *F*^2^, conventional *R*-factors *R* are based on *F*, with *F* set to zero for negative *F*^2^. The threshold expression of *F*^2^ > σ(*F*^2^) is used only for calculating *R*-factors(gt) *etc*. and is not relevant to the choice of reflections for refinement. *R*-factors based on *F*^2^ are statistically about twice as large as those based on *F*, and *R*- factors based on ALL data will be even larger.

### Fractional atomic coordinates and isotropic or equivalent isotropic displacement parameters (Å^2^)

**Table:**

	*x*	*y*	*z*	*U*_iso_*/*U*_eq_	Occ. (<1)
O1	0.5000	0.63088 (17)	0.8666 (3)	0.0557 (8)	
O2	0.5000	0.2129 (2)	0.5161 (3)	0.0585 (9)	
O3	0.5000	0.12875 (19)	0.3553 (3)	0.0584 (9)	
O4	0.5000	0.06903 (19)	0.5342 (3)	0.0608 (9)	
N1	0.5000	0.4061 (2)	0.9909 (3)	0.0415 (9)	
N2	0.5000	0.1632 (3)	0.9791 (4)	0.0451 (9)	
N3	0.5000	0.2207 (2)	0.7764 (3)	0.0447 (9)	
N4	0.5000	0.3731 (2)	0.7360 (4)	0.0499 (10)	
N5	0.5000	0.2581 (3)	1.1464 (4)	0.0524 (10)	
H1N5	0.5000	0.210 (3)	1.199 (4)	0.042 (13)*	
H2N5	0.5000	0.312 (3)	1.185 (5)	0.062 (14)*	
N6	0.5000	0.0703 (3)	0.8120 (4)	0.0546 (11)	
H1N6	0.5000	0.028 (3)	0.854 (5)	0.056 (15)*	
H2N6	0.5000	0.060 (2)	0.723 (3)	0.020 (9)*	
N7	0.5000	0.1359 (2)	0.4687 (4)	0.0448 (9)	
C1	0.5000	0.5648 (3)	0.9606 (5)	0.0467 (11)	
H1A	0.3776	0.5726	1.0120	0.056*	0.50
H1B	0.6224	0.5726	1.0120	0.056*	0.50
C2	0.5000	0.4726 (2)	0.9092 (4)	0.0390 (10)	
C3	0.5000	0.3228 (3)	0.9449 (4)	0.0384 (11)	
C4	0.5000	0.2451 (2)	1.0250 (4)	0.0401 (10)	
C5	0.5000	0.1519 (2)	0.8573 (5)	0.0408 (11)	
C6	0.5000	0.3074 (2)	0.8204 (5)	0.0391 (10)	
C7	0.5000	0.4546 (3)	0.7834 (4)	0.0498 (13)	
H7A	0.5000	0.5028	0.7293	0.060*	
H1O1	0.5000	0.689 (4)	0.901 (6)	0.072 (14)*	
H1N3	0.5000	0.208 (3)	0.683 (5)	0.046 (11)*	

### Atomic displacement parameters (Å^2^)

**Table:**

	*U*^11^	*U*^22^	*U*^33^	*U*^12^	*U*^13^	*U*^23^
O1	0.0954 (19)	0.0284 (13)	0.0435 (17)	0.000	0.000	0.0045 (16)
O2	0.090 (2)	0.0310 (14)	0.054 (2)	0.000	0.000	−0.0078 (16)
O3	0.096 (2)	0.0401 (17)	0.039 (2)	0.000	0.000	−0.0029 (17)
O4	0.093 (2)	0.0323 (15)	0.057 (2)	0.000	0.000	0.0045 (15)
N1	0.0534 (19)	0.0299 (17)	0.041 (2)	0.000	0.000	0.0027 (15)
N2	0.062 (2)	0.0276 (17)	0.046 (2)	0.000	0.000	0.0055 (15)
N3	0.068 (2)	0.0304 (18)	0.035 (2)	0.000	0.000	0.0005 (14)
N4	0.082 (3)	0.0300 (15)	0.038 (2)	0.000	0.000	0.0011 (17)
N5	0.086 (2)	0.035 (2)	0.036 (2)	0.000	0.000	0.0041 (18)
N6	0.082 (3)	0.0308 (18)	0.051 (3)	0.000	0.000	−0.001 (2)
N7	0.058 (2)	0.036 (2)	0.040 (2)	0.000	0.000	−0.0012 (17)
C1	0.069 (3)	0.034 (2)	0.037 (2)	0.000	0.000	0.0015 (19)
C2	0.056 (2)	0.032 (2)	0.029 (2)	0.000	0.000	0.0006 (18)
C3	0.046 (2)	0.027 (2)	0.041 (3)	0.000	0.000	0.0013 (15)
C4	0.049 (2)	0.032 (2)	0.040 (3)	0.000	0.000	0.007 (2)
C5	0.049 (2)	0.029 (2)	0.044 (3)	0.000	0.000	0.006 (2)
C6	0.050 (2)	0.0322 (19)	0.035 (2)	0.000	0.000	−0.0005 (18)
C7	0.075 (3)	0.036 (2)	0.038 (3)	0.000	0.000	0.003 (2)

### Geometric parameters (Å, º)

**Table:**

O1—C1	1.422 (6)	N5—C4	1.333 (7)
O1—H1O1	0.94 (6)	N5—H1N5	0.92 (5)
O2—N7	1.262 (5)	N5—H2N5	0.91 (5)
O3—N7	1.237 (5)	N6—C5	1.317 (5)
O4—N7	1.228 (5)	N6—H1N6	0.78 (5)
N1—C2	1.334 (5)	N6—H2N6	0.98 (4)
N1—C3	1.342 (5)	C1—C2	1.487 (6)
N2—C4	1.322 (5)	C1—H1A	0.9700
N2—C5	1.334 (7)	C1—H1B	0.9700
N3—C5	1.353 (6)	C2—C7	1.394 (5)
N3—C6	1.383 (5)	C3—C6	1.372 (6)
N3—H1N3	1.03 (5)	C3—C4	1.454 (5)
N4—C7	1.323 (5)	C7—H7A	0.9300
N4—C6	1.344 (6)		
			
C1—O1—H1O1	111 (4)	C2—C1—H1B	109.2
C2—N1—C3	116.4 (4)	H1A—C1—H1B	107.9
C4—N2—C5	119.5 (4)	N1—C2—C7	120.6 (3)
C5—N3—C6	119.3 (4)	N1—C2—C1	116.2 (4)
C5—N3—H1N3	120 (2)	C7—C2—C1	123.3 (3)
C6—N3—H1N3	121 (2)	N1—C3—C6	121.6 (4)
C7—N4—C6	114.1 (5)	N1—C3—C4	121.3 (4)
C4—N5—H1N5	120 (3)	C6—C3—C4	117.2 (4)
C4—N5—H2N5	126 (3)	N2—C4—N5	120.6 (3)
H1N5—N5—H2N5	114 (4)	N2—C4—C3	121.0 (5)
C5—N6—H1N6	122 (4)	N5—C4—C3	118.4 (4)
C5—N6—H2N6	121 (2)	N6—C5—N2	119.3 (4)
H1N6—N6—H2N6	117 (4)	N6—C5—N3	117.5 (5)
O4—N7—O3	120.5 (4)	N2—C5—N3	123.2 (4)
O4—N7—O2	120.4 (4)	N4—C6—C3	123.4 (4)
O3—N7—O2	119.0 (4)	N4—C6—N3	116.8 (4)
O1—C1—C2	112.0 (4)	C3—C6—N3	119.9 (4)
O1—C1—H1A	109.2	N4—C7—C2	124.1 (4)
C2—C1—H1A	109.2	N4—C7—H7A	118.0
O1—C1—H1B	109.2	C2—C7—H7A	118.0
			
C3—N1—C2—C7	0.000 (2)	C6—N3—C5—N6	180.000 (1)
C3—N1—C2—C1	180.000 (2)	C6—N3—C5—N2	0.000 (2)
O1—C1—C2—N1	180.000 (2)	C7—N4—C6—C3	0.000 (2)
O1—C1—C2—C7	0.000 (2)	C7—N4—C6—N3	180.000 (1)
C2—N1—C3—C6	0.000 (2)	N1—C3—C6—N4	0.000 (2)
C2—N1—C3—C4	180.000 (2)	C4—C3—C6—N4	180.000 (2)
C5—N2—C4—N5	180.000 (2)	N1—C3—C6—N3	180.000 (2)
C5—N2—C4—C3	0.000 (2)	C4—C3—C6—N3	0.000 (2)
N1—C3—C4—N2	180.000 (2)	C5—N3—C6—N4	180.000 (1)
C6—C3—C4—N2	0.000 (2)	C5—N3—C6—C3	0.000 (2)
N1—C3—C4—N5	0.000 (2)	C6—N4—C7—C2	0.000 (2)
C6—C3—C4—N5	180.000 (2)	N1—C2—C7—N4	0.000 (2)
C4—N2—C5—N6	180.000 (2)	C1—C2—C7—N4	180.000 (2)
C4—N2—C5—N3	0.000 (2)		

### Hydrogen-bond geometry (Å, º)

**Table:**

*D*—H···*A*	*D*—H	H···*A*	*D*···*A*	*D*—H···*A*
N3—H1*N*3···O2	1.03 (5)	1.82 (5)	2.831 (5)	167 (4)
N6—H2*N*6···O4	0.98 (3)	2.06 (3)	3.019 (6)	167 (3)
O1—H1*O*1···O2^i^	0.95 (6)	1.93 (6)	2.846 (4)	163 (6)
N5—H1*N*5···O3^ii^	0.92 (4)	2.09 (4)	2.983 (6)	164 (4)
N5—H2*N*5···O1^i^	0.91 (5)	2.15 (5)	2.913 (5)	141 (4)
N6—H1*N*6···O3^iii^	0.78 (5)	2.35 (4)	3.015 (5)	145 (5)
N6—H1*N*6···O4^iii^	0.78 (5)	2.44 (5)	3.190 (6)	162 (5)

Symmetry codes: (i) −*x*+1, −*y*+1, *z*+1/2; (ii) *x*, *y*, *z*+1; (iii) −*x*+1, −*y*, *z*+1/2.
