# Supplementary figures and images for: Crystal structure of 2,4-di­amino-7-(hydroxy­meth­yl)pteridin-1-ium nitrate
Source: Acta Crystallogr E Crystallogr Commun. 2015 May 7;71(Pt 6):o376–7. doi: 10.1107/S2056989015008397 (PMC4459323; doi:10.1107/S2056989015008397)

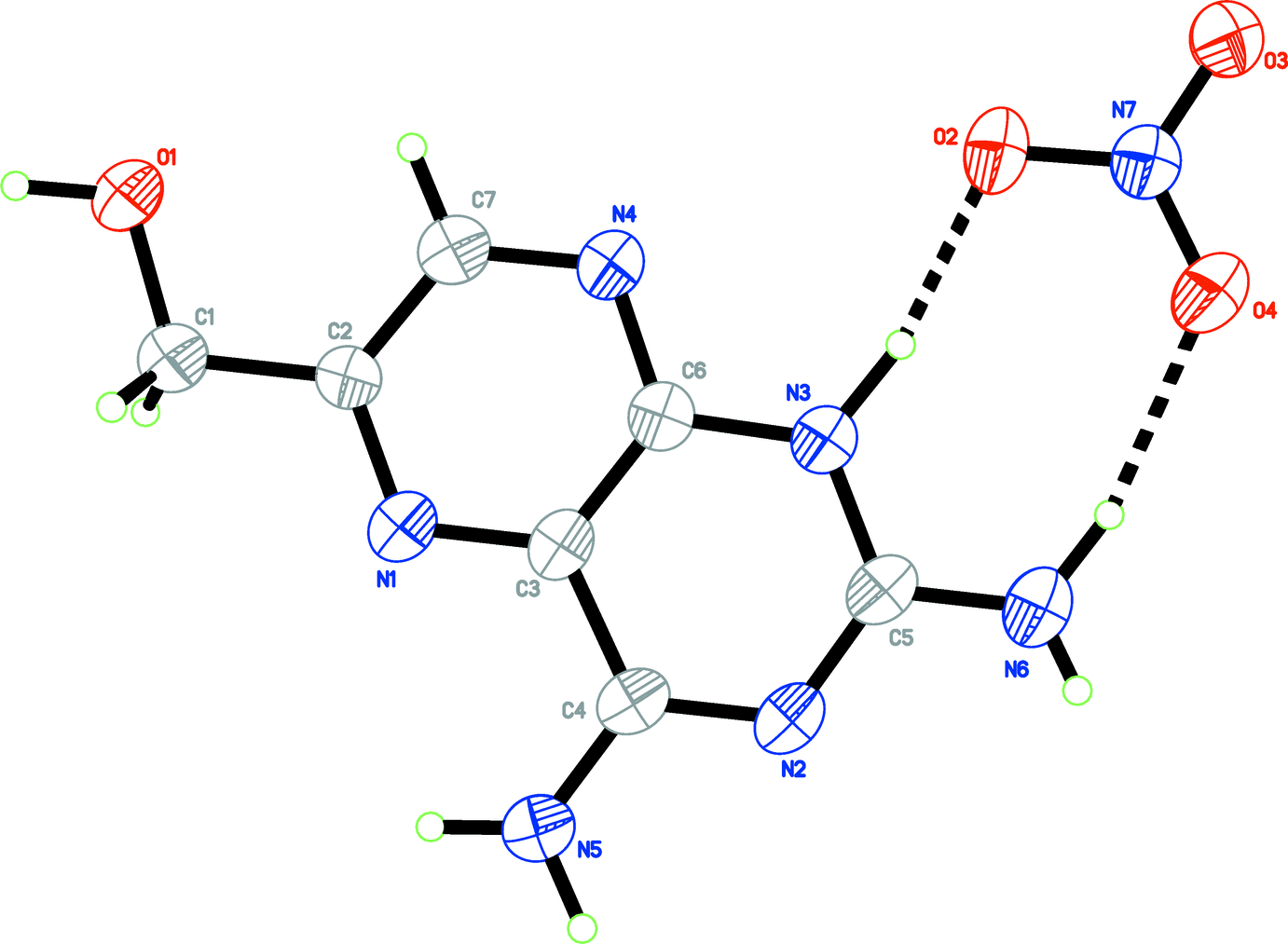

Supplement: Supplementary file 4 [file e-71-0o376-fig1.tif]

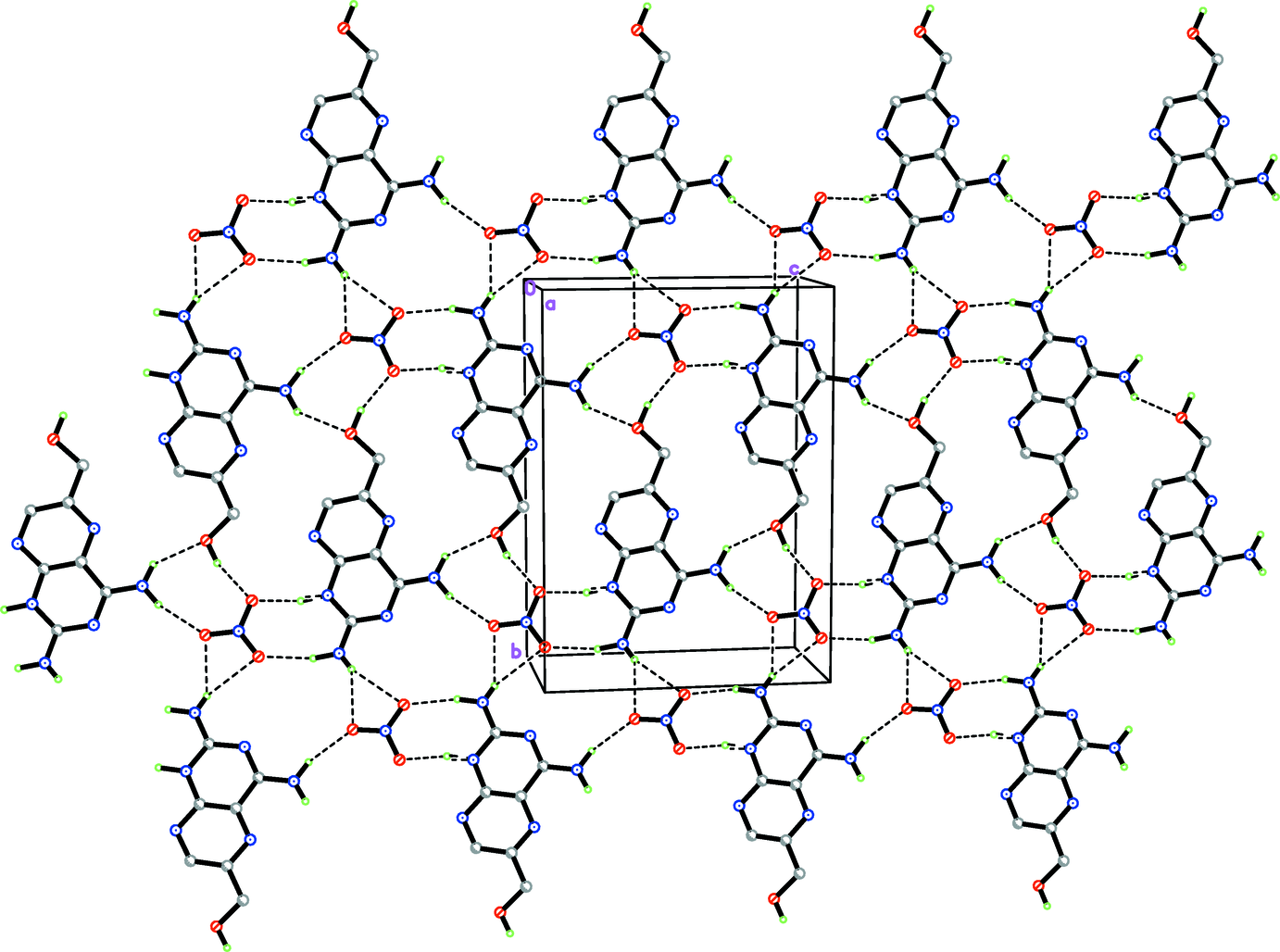

Supplement: Supplementary file 5 [file e-71-0o376-fig2.tif]
